# The Potential Impact of a Hepatitis C Vaccine for People Who Inject Drugs: Is a Vaccine Needed in the Age of Direct-Acting Antivirals?

**DOI:** 10.1371/journal.pone.0156213

**Published:** 2016-05-25

**Authors:** Jack Stone, Natasha K. Martin, Matthew Hickman, Margaret Hellard, Nick Scott, Emma McBryde, Heidi Drummer, Peter Vickerman

**Affiliations:** 1 School of Social and Community Medicine, University of Bristol, Bristol, United Kingdom; 2 Division of Global Public Health, University of California San Diego, La Jolla, United States of America; 3 Centre for Population Health, Burnet Institute, Melbourne, Australia; 4 Centre for Biomedical Research, Burnet Institute, Melbourne, Australia; Saint Louis University, UNITED STATES

## Abstract

**Background and Aims:**

The advent of highly effective hepatitis C (HCV) treatments has questioned the need for a vaccine to control HCV amongst people who inject drugs (PWID). However, high treatment costs and ongoing reinfection risk suggest it could still play a role. We compared the impact of HCV vaccination amongst PWID against providing HCV treatment.

**Methods:**

Dynamic HCV vaccination and treatment models among PWID were used to determine the vaccination and treatment rates required to reduce chronic HCV prevalence or incidence in the UK over 20 or 40 years. Projections considered a low (50% protection for 5 years), moderate (70% protection for 10 years) or high (90% protection for 20 years) efficacy vaccine. Sensitivities to various parameters were examined.

**Results:**

To halve chronic HCV prevalence over 40 years, the low, moderate and high efficacy vaccines required annual vaccination rates (coverage after 20 years) of 162 (72%), 77 (56%) and 44 (38%) per 1000 PWID, respectively. These vaccination rates were 16, 7.6 and 4.4 times greater than corresponding treatment rates. To halve prevalence over 20 years nearly doubled these vaccination rates (moderate and high efficacy vaccines only) and the vaccination-to-treatment ratio increased by 20%. For all scenarios considered, required annual vaccination rates and vaccination-to-treatment ratios were at least a third lower to reduce incidence than prevalence. Baseline HCV prevalence had little effect on the vaccine’s impact on prevalence or incidence, but substantially affected the vaccination-to-treatment ratios. Behavioural risk heterogeneity only had an effect if we assumed no transitions between high and low risk states and vaccinations were targeted or if PWID were high risk for their first year.

**Conclusions:**

Achievable coverage levels of a low efficacy prophylactic HCV vaccine could greatly reduce HCV transmission amongst PWID. Current high treatment costs ensure vaccination could still be an important intervention option.

## Introduction

Hepatitis C virus (HCV) is a blood-borne disease with an estimated 80 million viraemic infections worldwide[1]. HCV infection can result in liver cirrhosis[2], with associated risks of liver failure and hepatocellular carcinoma.

In developed countries, the primary mode of transmission is through injecting drug use[3]. Globally, HCV sero-prevalence amongst people who inject drugs (PWID) is 60%[4]. Prevention of HCV among PWID is critical to reducing HCV transmission and HCV-related morbidity.

Needle and syringe programmes (NSPs) and opiate substitution therapy (OST) can reduce the risk of acquiring HCV[5] but may be insufficient for reducing HCV prevalence to low levels[6]. Modeling has shown the potential benefits of HCV treatment as prevention, particularly with new highly effective direct-acting antiviral therapies (DAAs)[7, 8]. However, the high costs of these treatments (>$85,000 and $54,000 for a 12-week course of Sofosbuvir in the USA and UK respectively[9] and $94,500 for a 12-week course of Harvoni[10]) currently restricts their widespread use for prevention.

An alternative prevention strategy could be vaccination. Early HCV vaccine studies in chimpanzees found that a vaccine eliciting a high efficacy humoral response effectively controlled and facilitated clearance of the homologous HCV genotype 1a challenge virus[11]. Phase I studies conducted in humans demonstrated that the vaccine was safe and well tolerated and that some individuals elicited broadly reactive neutralizing antibodies (NAbs)[12], although development of this vaccine has since stalled because HCV has numerous immune evasion strategies that limit the effectiveness of the NAb response[13]. An alternative approach that generates T-cell responses has also been tested in human phase 1 studies and was well tolerated and highly immunogenic[14]. Ongoing phase 2 trials amongst PWID are determining the efficacy of this vaccine[15]. Thus, a partially effective prophylactic HCV vaccine could soon become available. It is, therefore, important to assess the possible utility of HCV vaccination for HCV prevention and to compare its impact against new highly effective DAAs.

## Methods

We developed a dynamic deterministic HCV transmission model among PWID incorporating prophylactic vaccination to determine the annual vaccination rates required to reduce chronic prevalence and incidence amongst PWID by 25, 50 and 75% over 20 and 40 years. Using a HCV treatment model[7], we determined the corresponding annual treatment rates that achieve the same impact and calculate the corresponding ratio of the vaccination to treatment rates.

### Mathematical model: vaccine

**Fig 1:**
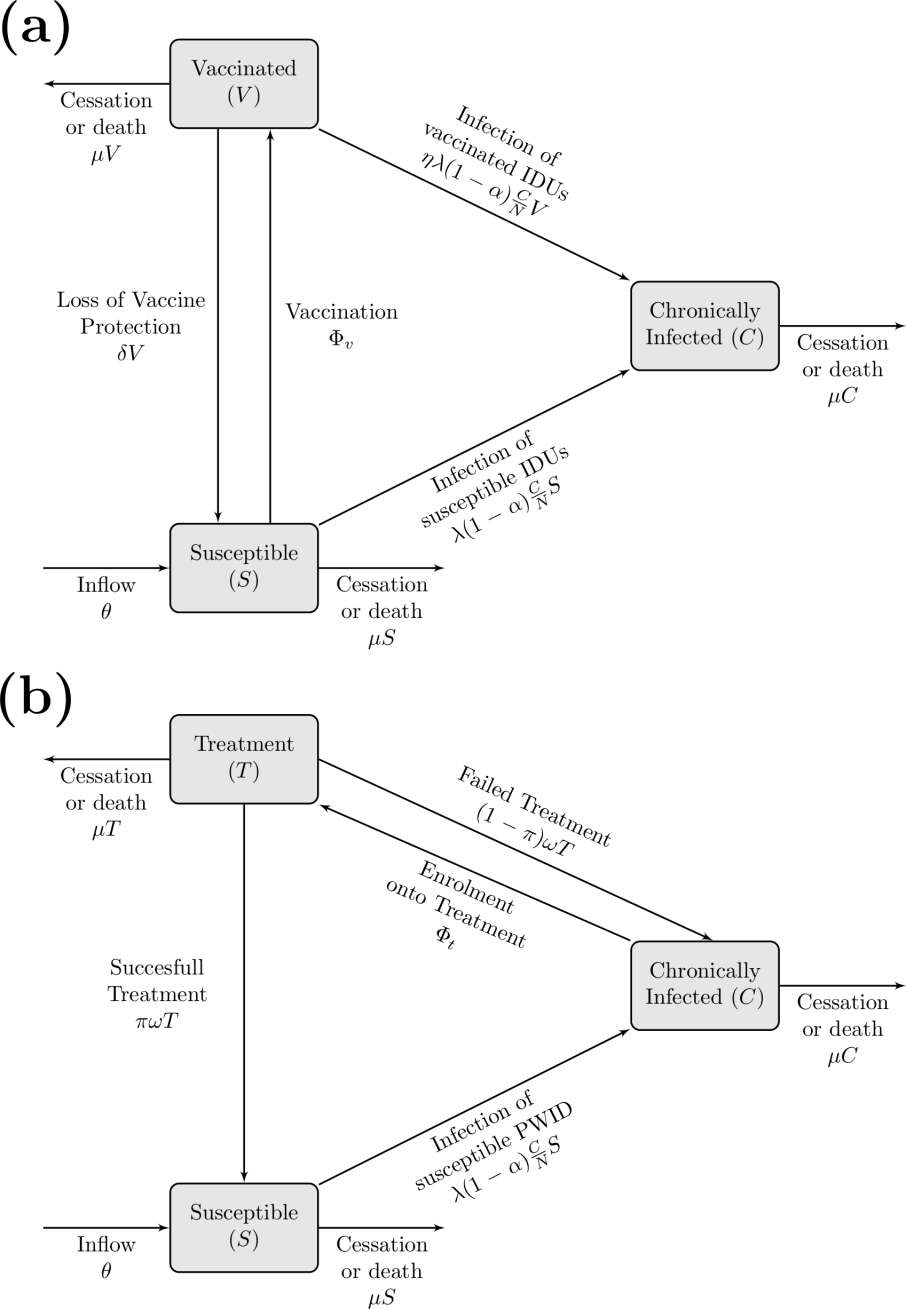
Model Schematics. Schematic for the mathematical models including vaccination (a) and treatment (b). The parameters used are defined in Table 1.

The vaccine model includes three groups of PWID (Fig 1A): susceptible (including spontaneously cleared infections), chronically infected, and vaccinated (model equations in S1 File). Acute infection was not included because previous modelling suggests it contributes little to transmission[6, 7].

**Table 1:** Model Parameters and Sources.

*Parameter*		*Symbol*	*Value at Baseline*	*Models*	*Source*
PWID leaving rate (cessation + death) per year		μ	0.085^a^	Both	[19, 20]
Inflow rate of new injectors per year (per 1000 PWID)		θ	85^b^	Both	…
Infection rate per year		λ	0.191^c^	Both	…
Initial chronic prevalence (%)		…	40	Both	[17, 18]
Proportion of new infections that spontaneously clear		α	0.26	Both	[18]
PWID vaccinated per 1000 per year		Φ_v_	varied	Vaccine	…
PWID treated per 1000 per year		Φ_t_	varied	Treatment	…
Duration of protection from vaccine (years)		1/δ		Vaccine	…
	‘Low efficacy’ vaccine		**5**		
	‘Moderate’ vaccine		10		
	‘High efficacy’ vaccine		20		
Efficacy of vaccine (%)		100(1-η)		Vaccine	…
	‘Low efficacy’ vaccine		50		
	‘Moderate’ vaccine		70		
	‘High efficacy’ vaccine		90		
Proportion of infections cured with treatment		ᴨ	90	Treatment	[21]
Duration of treatment (weeks)		52/ω	12	Treatment	[22]

a Based on a cessation rate of 7.75% per year, and a PWID death rate of 0.75%

b Set to be 1000μ in order to maintain a total population size of 1000 PWID

c Varied to fit chronic prevalence

New PWID enter the susceptible pool at fixed rate θ and leave all compartments either through death or permanent cessation of injection (rate μ). The model is dynamic, with the rate of infection of susceptible PWID being proportional to the prevalence of chronic infection and infection rate, λ. Acute infection spontaneously clears in a proportion, α, who remain susceptible. The remaining infected fraction, (1—α) progress to chronic infection.

A fixed number (Φ_v_) of susceptible (RNA-negative) PWID are vaccinated annually unless the number of susceptible PWID is below Φ_v_, whereupon all susceptible PWID are vaccinated. PWID remain in the vaccinated state for an average duration 1\δ where they experience a factor η lower infection rate than susceptible PWID, giving 100(1-η)% as the degree to which the vaccine reduces the risk of HCV acquisition or the vaccine efficacy. PWID losing protection from the vaccine re-enter the susceptible pool, and can be revaccinated without a loss in protection.

### Mathematical model: treatment

Fig 1B shows the model schema[7] used to project the prevention impact of IFN-free DAAs (model equations in S1 File). The model dynamics for entry, exit and infection are identical to the vaccine model, with the only model difference being the intervention component. In this model, a fixed number (Φ_t_) of chronically infected PWID are treated each year. If the treatment rate exceeds the number of chronically infected PWID, then all are treated. PWID undergoing treatment are assumed to be non-infectious due to rapid reductions in viral load when on treatment[16] and, given the different treatment options available, PWID failing treatment are eligible for re-treatment without any loss in sustained viral response (SVR ᴨ). No immunity is assumed because previous analyses showed it had little effect[6, 7].

### Simulations and model parameters

Since most current HCV epidemics are not in the epidemic phase, we allowed the models to reach a stable endemic state prior to initiating vaccination or treatment. Parameter values were obtained from relevant literature (Table 1). The model was parameterised to a UK baseline scenario where 40% of PWID have chronic HCV[17, 18], with the leaving rate *μ* including cessation of injecting (7.75% per year) and death (0.75% per year)[19, 20].

### Baseline impact projections for the UK

To investigate the impact of vaccination in the UK, three vaccines were considered: a ‘low efficacy’ vaccine that provides 50% protection against acquisition of infection for 5 years; a ‘moderate efficacy’ vaccine that provides 70% protection for 10 years; and a ‘high efficacy’ vaccine that provides 90% protection for 20 years. For each vaccine, we projected the reduction in HCV chronic prevalence over time for different vaccination rates and the annual vaccination rates needed to reduce prevalence and incidence by 25, 50 or 75% over 20 or 40 years.

### Comparing the impact of vaccination with treatment

For the baseline UK scenario, the HCV treatment model was used to determine the annual treatment rate required to reduce prevalence and incidence by 25, 50 and 75% after 20 or 40 years. These treatment rates were used to calculate the vaccination-to-treatment ratio, i.e. the number of vaccinations needed per treatment to achieve the same impact; either on HCV chronic prevalence or incidence. These treatment projections assumed parameters corresponding to DAA treatments (Table 1).

### Sensitivity analyses

For the baseline UK scenario, we performed univariate sensitivity analyses on the vaccination rate needed for the moderate efficacy vaccine to halve chronic prevalence and incidence after 40 years. By undertaking parallel sensitivity analyses with the treatment model, we also determined the robustness of the vaccination-to-treatment ratios.

The sensitivity analyses explored how changes in initial HCV prevalence, injecting duration, vaccine efficacy and duration of protection affected the required vaccination rates or vaccination-to-treatment ratios.

We also considered the effect of including heterogeneity in HCV transmission risk amongst PWID. Half of PWID were assumed to be high-risk corresponding to the proportion of PWID injecting crack or currently homeless in the UK[5], which were assumed to have 2 or 6 times the HCV transmission risk of low-risk PWID[5, 6]. We explored the effect of no transitioning between the risk groups or an average duration high-risk of 2 years[6] with low-risk PWID transitioning to high-risk to balance this flow. We also consider a scenario where PWID are at higher risk of HCV infection in their first year of injecting such that the prevalence amongst recent PWID is half that of non-recent PWID[23]. All scenarios incorporating risk heterogeneity assumed random mixing because moderate like-with-like mixing has little effect[6, 24].

Lastly, we considered the implications of assuming immunity follows spontaneous clearance of infection, modelled by assuming that PWID who spontaneously clear infection have the same protection from reinfection as vaccinated PWID; i.e. 70% protection for 10 years.

## Results

### UK impact projections: vaccine

**Fig 2:**
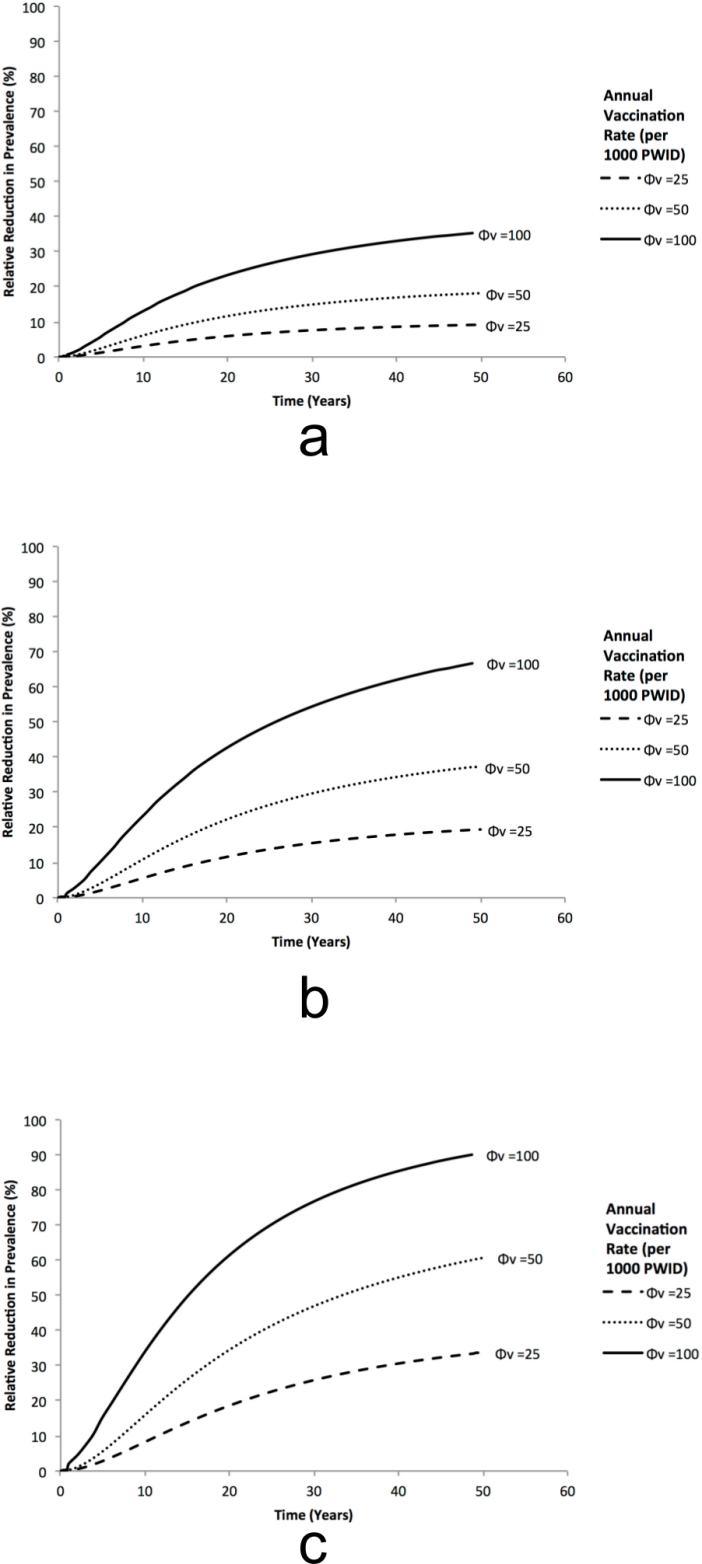
Impact Projections for HCV Vaccination. Projected relative reduction in chronic HCV prevalence among PWID in the UK over time for various vaccination rates (Φ_v_ per 1000 PWID per year) with vaccines that have (A) low: 50% protection for 5 years, (B) moderate: 70% protection for 10 years, or (C) high efficacy: 90% protection for 20 years.

For the baseline UK scenario (40% chronic prevalence), annual vaccination rates of 100 per 1000 PWID could achieve a relative prevalence reduction among PWID of 35%, 65%, or 85% after 40 years for the low (50% protection for 5 years), moderate (70% protection for 10 years), and high efficacy (90% protection for 20 years) vaccines, respectively (Fig 2). These vaccination rates result in a 61% vaccination coverage (proportion of current PWID who have ever been vaccinated) after 20 years for the low efficacy vaccine, but higher coverage levels for the moderate (68%) and high efficacy (75%) vaccines due to less re-vaccination. Vaccination rates below 50 per 1000 PWID per year will have modest impact (20% reduction in 50 years) with a low efficacy vaccine, but could halve prevalence in 30 years with a high efficacy vaccine. For all scenarios, close to maximum impact is achieved after 50 years (S1 Fig).

**Fig 3:**
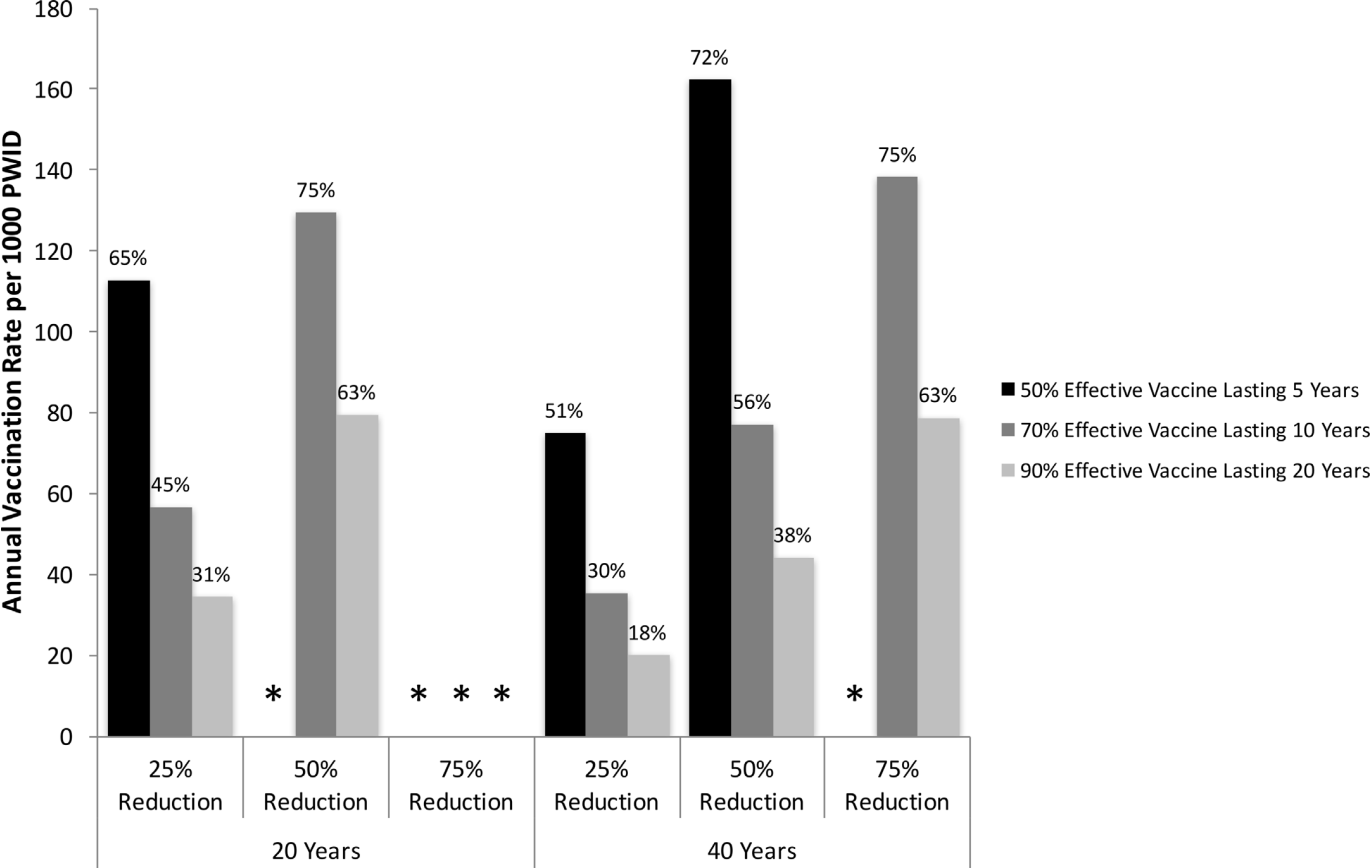
Vaccination rates required to reduce HCV prevalence. Annual vaccination rate (per 1000 PWID) and coverage levels (after 20 years–shown on top of bars) needed to reduce UK HCV chronic prevalence among PWID by a relative 25, 50 and 75% over 20 or 40 years, for a low (50% protection for 5 years), moderate (70% protection for 10 years) or high efficacy vaccine (90% protection for 20 years). Asterisks mark scenarios in which vaccine cannot achieve desired reduction in prevalence over time period.

Fig 3 shows that the low efficacy vaccine cannot halve prevalence over 20 years, but can over 40 years with high annual vaccination rates (162 per 1000 PWID) and resulting vaccination coverage (72%). Higher efficacy vaccines require much lower vaccination rates or coverage levels (44 per 1000 PWID annually or 38% respectively for the high efficacy vaccine) to achieve the same impact over 40 years. Greater reductions can also be achieved; the moderate and high efficacy vaccines can reduce prevalence by 75% over 40 years with annual vaccination rates of 138 and 79 per 1000 PWID, respectively. However, for the moderate vaccine (only scenario where the vaccination rate is not maintained), this vaccination rate requires all susceptible PWID to be vaccinated between years 7 and 21. For all vaccines considered, the vaccination rate needed to halve prevalence is more than twice what is needed to reduce prevalence by 25% over the same time period, and is nearly doubled again to reduce prevalence by 75%. Conversely, as the follow-up time is increased from 20 to 40 years, the annual number of vaccinations needed to achieve the same impact decreases by up to 44%.

**Fig 4:**
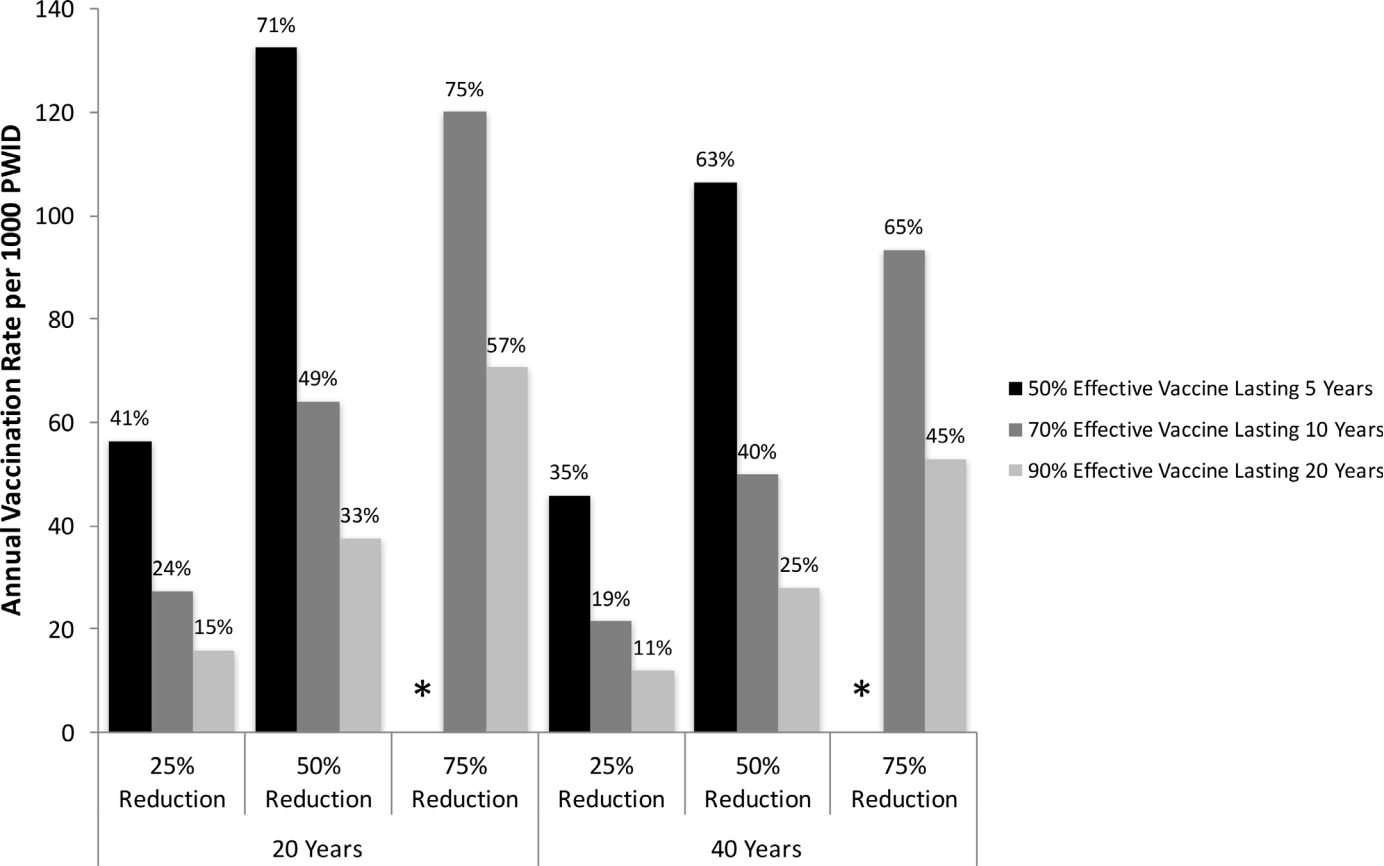
Vaccination rates required to reduce HCV incidence. Annual vaccination rate (per 1000 PWID) and coverage levels (after 20 years–shown on top of bars) needed to reduce UK HCV chronic incidence among PWID by a relative 25, 50 and 75% over 20 or 40 years, for a low (50% protection for 5 years), moderate (70% protection for 10 years) or high efficacy vaccine (90% protection for 20 years). Asterisks mark scenarios in which vaccination cannot achieve desired reduction in incidence over time period.

For all vaccines and impact levels considered, the required vaccination rates needed to reduce HCV incidence over both 20 and 40 years (Fig 4) are smaller than that needed to reduce prevalence to the same degree (Fig 3), suggesting that vaccination achieves reductions in incidence faster than prevalence. For example, with the moderate vaccine, vaccination rates of 64 and 129 per 1000 PWID are needed to halve incidence and prevalence, respectively, over 20 years, and these reduce to 50 and 77 per 1000 PWID to halve incidence and prevalence over 40 years. Furthermore, for some of the scenarios considered, vaccination can achieve reductions in incidence which cannot be achieved in prevalence. For example, the moderate efficacy vaccine could halve incidence over 20 years, with a vaccination rate of 120 per 1000 PWID, but could not halve prevalence over the same timespan.

#### Sensitivity analysis

**Fig 5:**
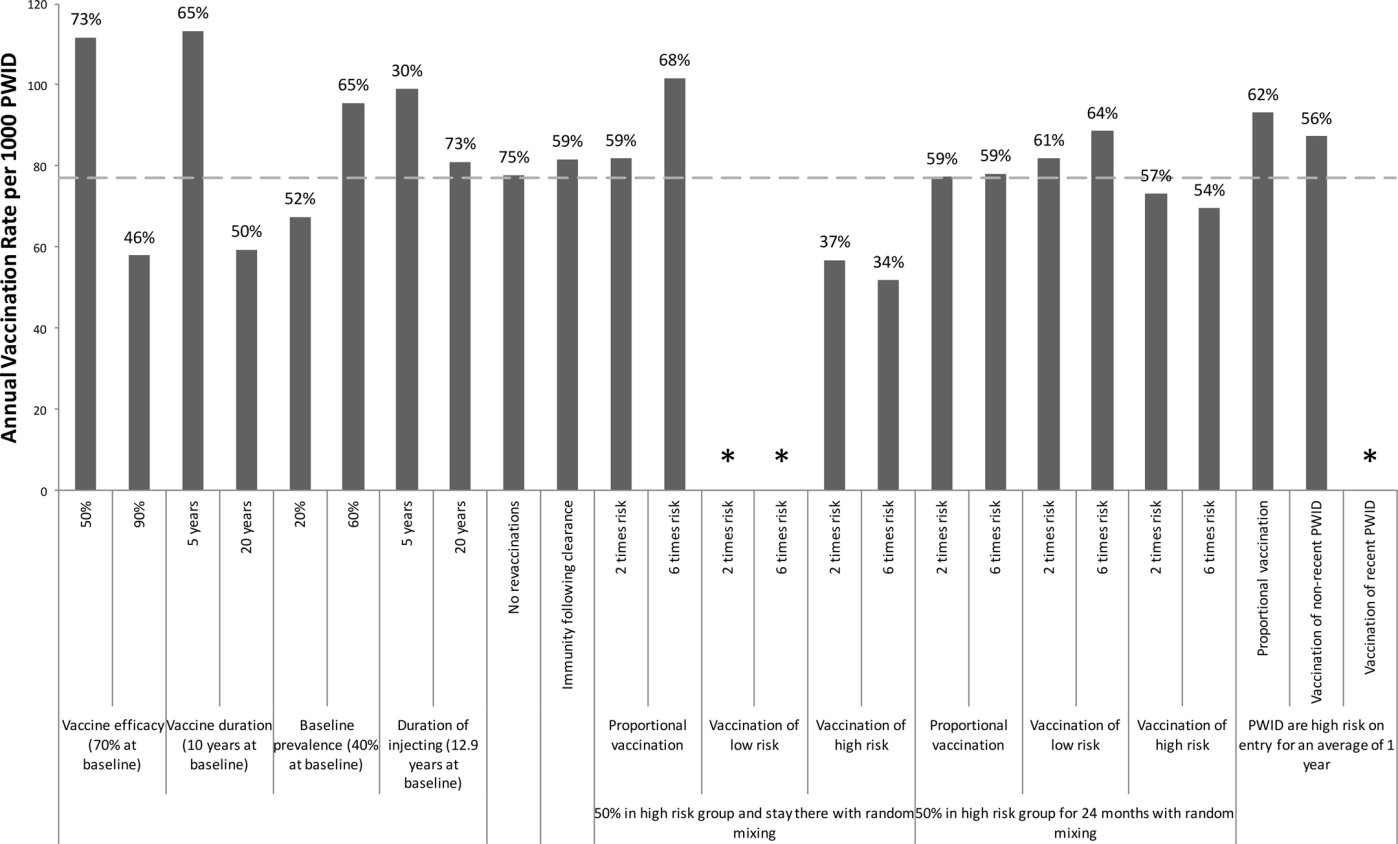
Sensitivity Analysis: Vaccination rates required to reduce HCV prevalence. One-way sensitivity analyses on how specific changes in model parameters affect the required annual vaccination rate per 1000 PWID (coverage level after 20 years shown above bars) for halving prevalence **among PWID** over 40 years in the UK. The baseline scenario (dashed line) involves the moderate efficacy vaccine with 70% protection for 10 years, and assumes no behavioural risk heterogeneity. Other baseline parameters are shown in the figure. Asterisks mark scenarios in which the vaccine cannot halve prevalence over 40 years.

**Fig 6:**
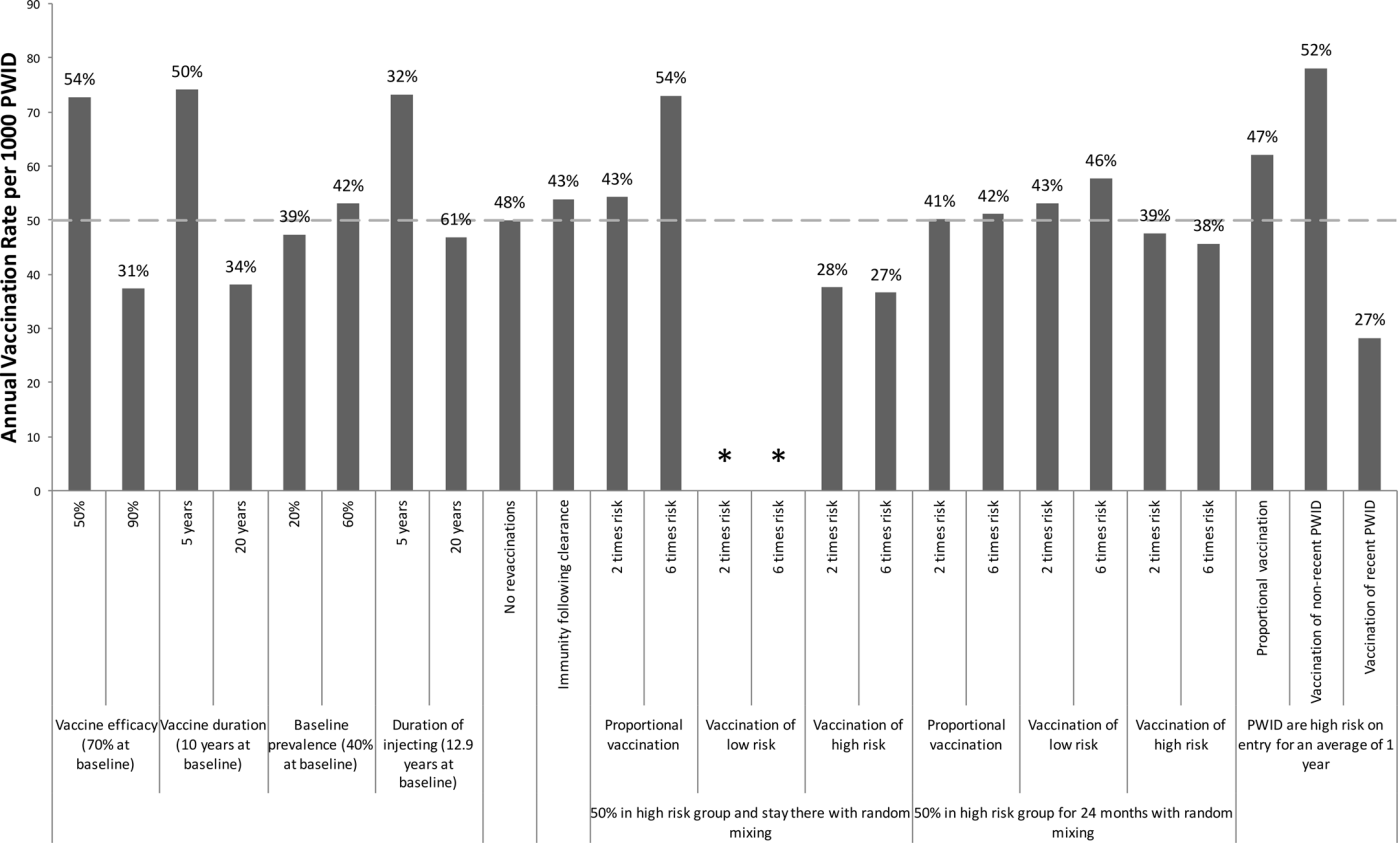
Sensitivity Analysis: Vaccination rates required to reduce HCV incidence. One-way sensitivity analyses on how specific changes in model parameters affect the required annual vaccination rate per 1000 PWID (coverage level after 20 years shown above bars) for halving incidence **among PWID** over 40 years in the UK. The baseline scenario (dashed line) involves the moderate efficacy vaccine with 70% protection for 10 years, and assumes no behavioural risk heterogeneity. Other baseline parameters are shown in the figure. Asterisks mark scenarios in which the vaccine cannot halve incidence over 40 years.

The sensitivity analysis (Fig 5) shows that the required vaccination rate to halve chronic prevalence over 40 years is not sensitive to variations in chronic prevalence or the duration of injecting when varied from 10 to 5 or 20 years, although larger deviations (S2 Fig) have greater effect. However, the duration of injecting has greater impact on the vaccination rate needed to halve incidence over 40 years; with the vaccination rate increasing by 46% if the duration of injecting was 5 years (Fig 6). As expected, the required vaccination rates to halve prevalence and incidence are sensitive to the vaccine’s duration of protection. Indeed, for all vaccine efficacies considered, the impact on prevalence appears to be a logarithmic function of duration of protection (S3 Fig). Additionally, not allowing revaccination has little effect except for vaccines with short durations of protection. Including immunity following spontaneous clearance of infection has little impact, increasing the required vaccination rates to halve prevalence or incidence by a relative 6 or 8%, respectively.

Incorporating behavioural risk heterogeneity also has little effect if the model includes transitions between risk groups. However, if there is no transition between risk groups then the required vaccination rates to halve chronic prevalence and incidence over 40 years increase considerably (by up to 46%) if vaccinations are distributed randomly, and decrease substantially (by up to 1/3) if high-risk PWID are targeted, while the desired prevalence and incidence reductions are not achievable if only low-risk PWID are vaccinated. Conversely, if recent PWID are higher-risk than non-recent PWID (9.4 times), then the required vaccination rates are slightly higher (<24%) than at baseline if vaccination is distributed randomly.

### Comparing the impact of vaccination to HCV treatment

**Fig 7:**
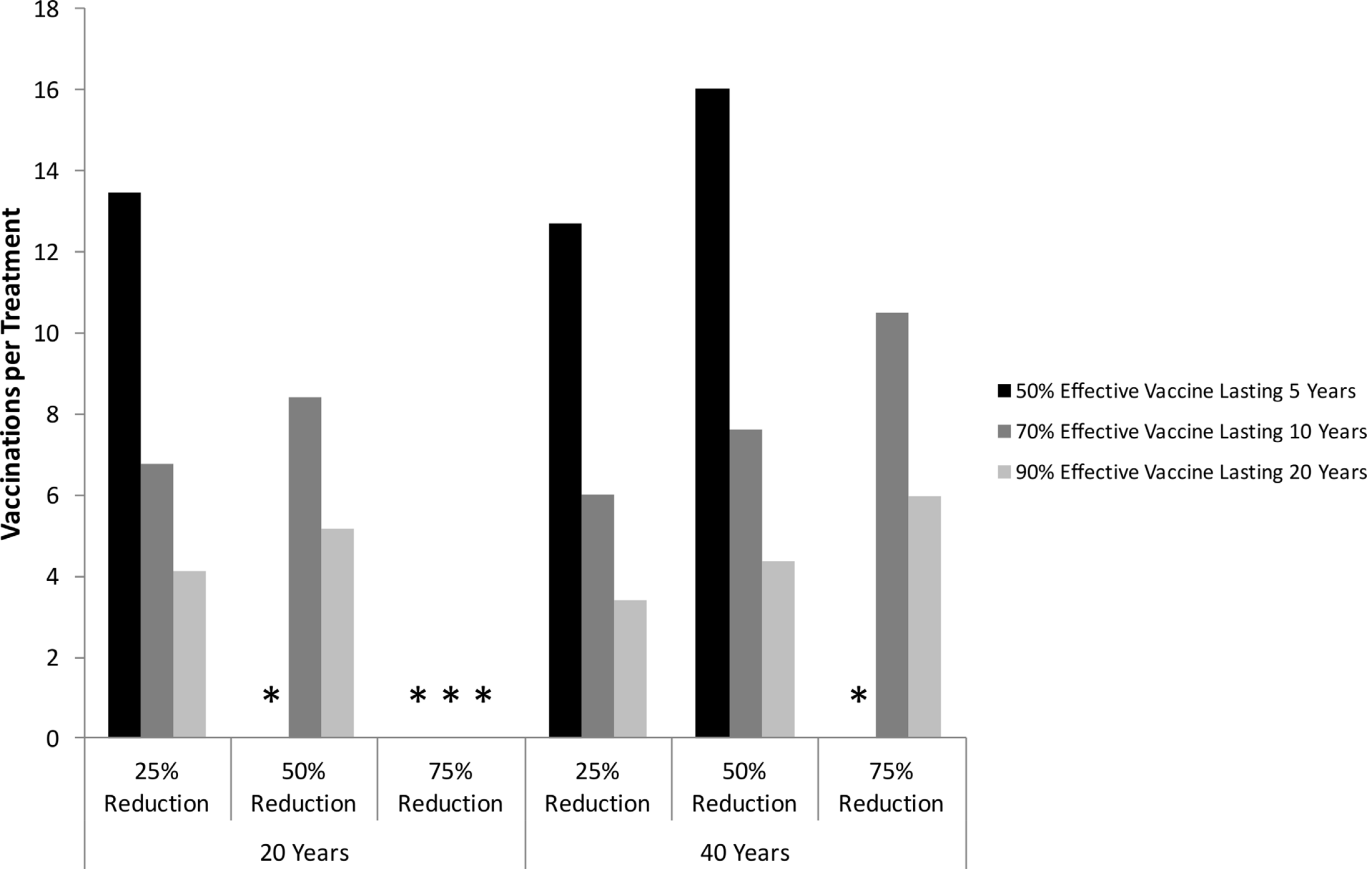
Vaccination-to-treatment ratios for reducing HCV prevalence. The number of vaccinations required per treatment for treatment and vaccination to have the same impact on prevalence **among PWID** in the UK (25/50/75% reduction) over 20 or 40 years, for three different vaccine scenarios: low (50% protection for 5 years); moderate (70% protection for 10 years); or high efficacy (90% protection for 20 years) Asterisks mark scenarios in which the vaccine cannot achieve the desired reduction in HCV chronic prevalence over that time period.

Fig 7 shows that the vaccination rate required to achieve a 25% reduction in prevalence over 40 years is 3.4, 6 or 13 times greater than the treatment rate needed for the same impact, for the high, moderate or low efficacy vaccines, respectively. These ratios increase for greater reductions in prevalence but decrease for increased follow-up times. For instance, the moderate efficacy vaccine needs 8.4 and 7.6 vaccinations per treatment to halve prevalence over 20 and 40 years, respectively, and over 10 to reduce prevalence by 75% over 40 years.

**Fig 8:**
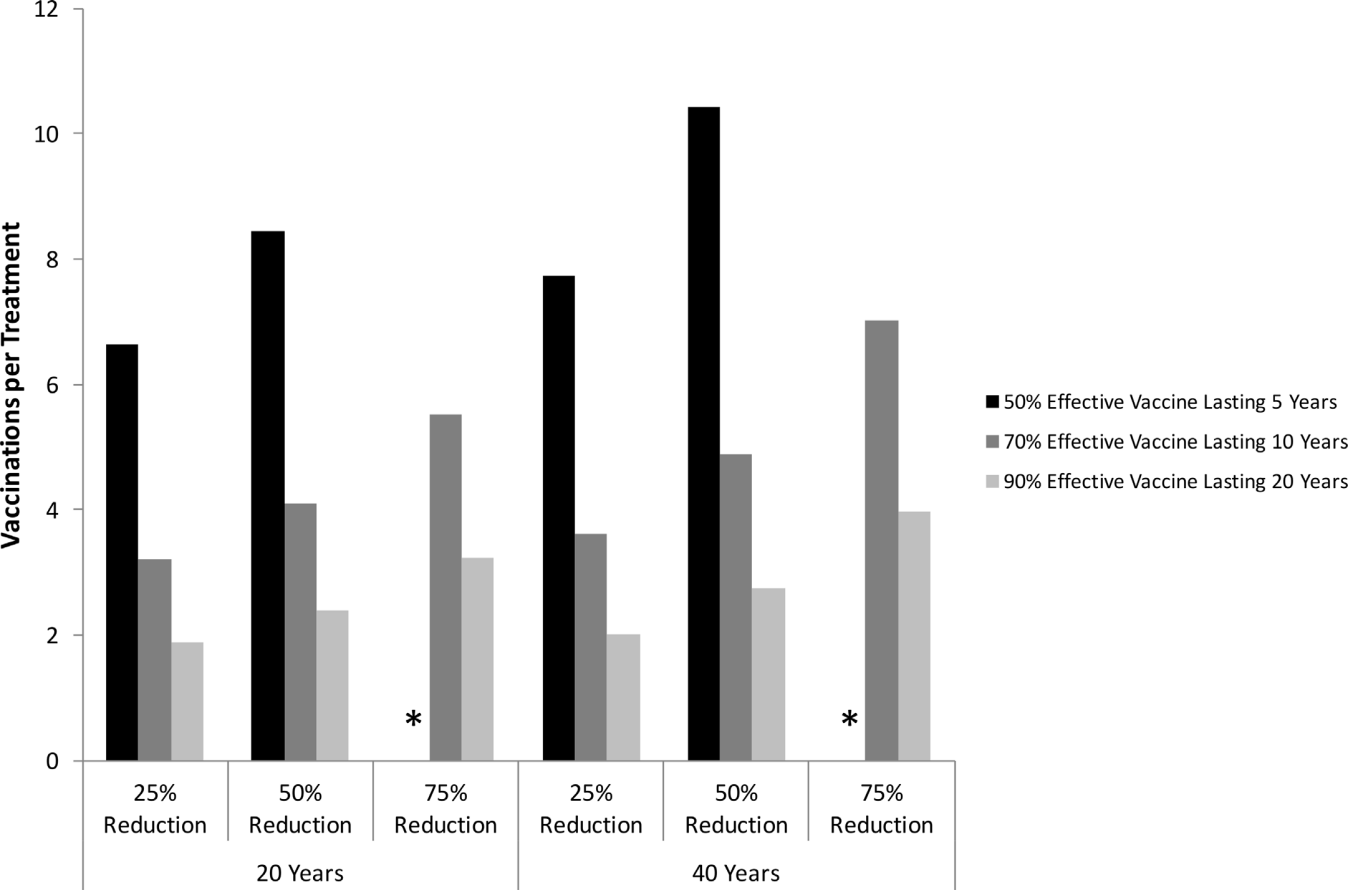
Vaccination-to-treatment ratios for reducing HCV incidence. The number of vaccinations required per treatment for treatment and vaccination to have the same impact on incidence **among PWID** in the UK (25/50/75% reduction) over 20 or 40 years, for three different vaccine scenarios: low (50% protection for 5 years); moderate (70% protection for 10 years); or high efficacy (90% protection for 20 years) Asterisks mark scenarios in which the vaccine cannot achieve the desired reduction in HCV chronic incidence over that time period.

When considering the impacts on incidence instead of prevalence, the vaccination-to-treatment ratios are much lower, with only 5 vaccinations per treatment being needed to halve incidence over 40 years for the moderate efficacy vaccine (Fig 8). Similar results are found for other decreases in incidence, with much lower (by at least a third) vaccination-to-treatment ratios being needed to reduce incidence rather than prevalence. However, in all scenarios considered, the vaccination-to-treatment ratios favor treatment, with the lowest ratio (1.9) observed when considering the vaccination rate required to reduce incidence by 25% over 20 years with the high efficacy vaccine.

#### Sensitivity analysis

**Fig 9:**
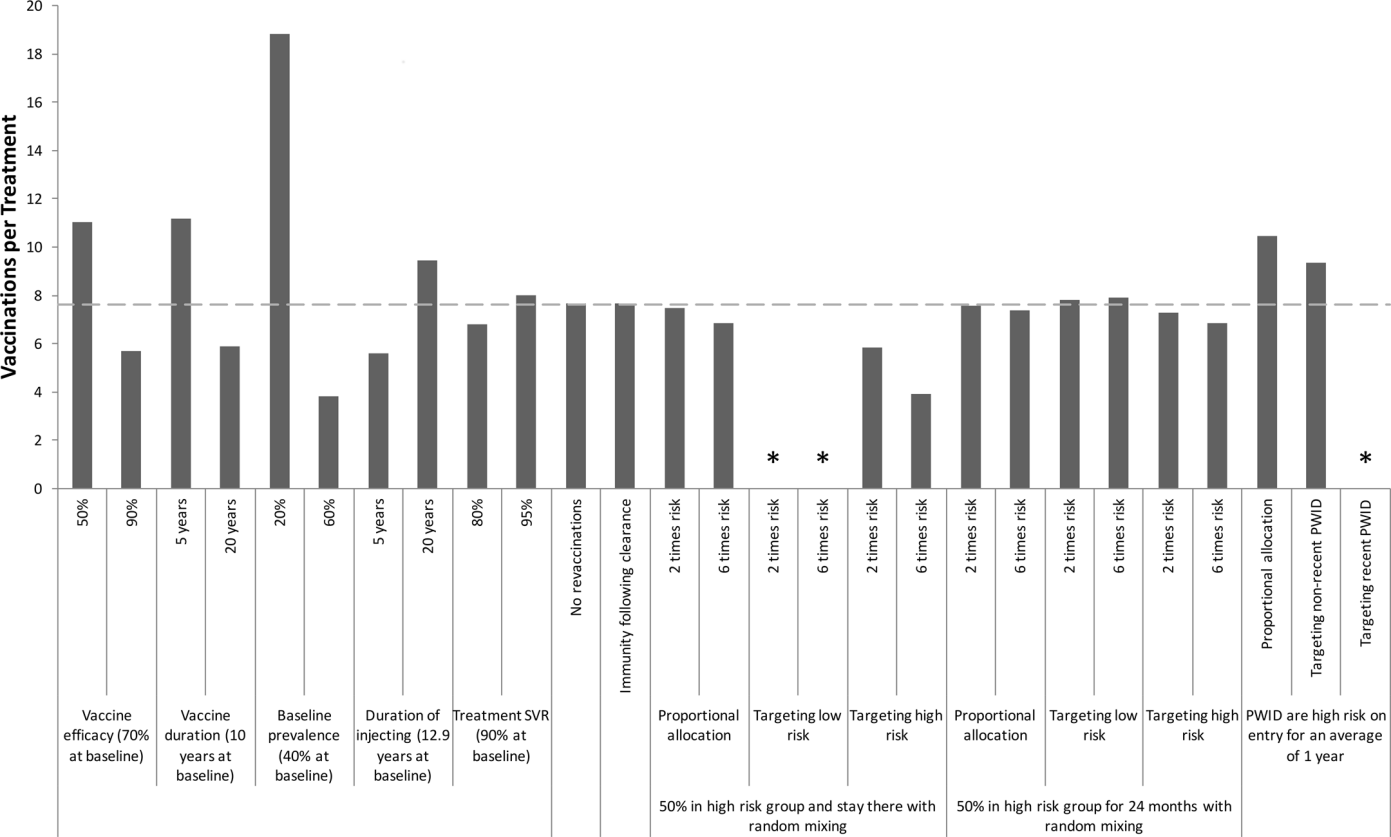
Sensitivity Analysis: Vaccination-to-treatment ratios for reducing HCV prevalence. One-way sensitivity analyses on how specific changes in model parameters affect the ratio of the required annual vaccination to treatment rate needed to halve prevalence **among PWID** over 40 years in the UK for a vaccine with 70% efficacy and duration of protection of 10 years. The baseline scenario (dashed line) assumed no behavioural risk heterogeneity with other baseline parameters shown in the figure. Asterisks mark scenarios in which the vaccine cannot halve prevalence over 40 years.

**Fig 10:**
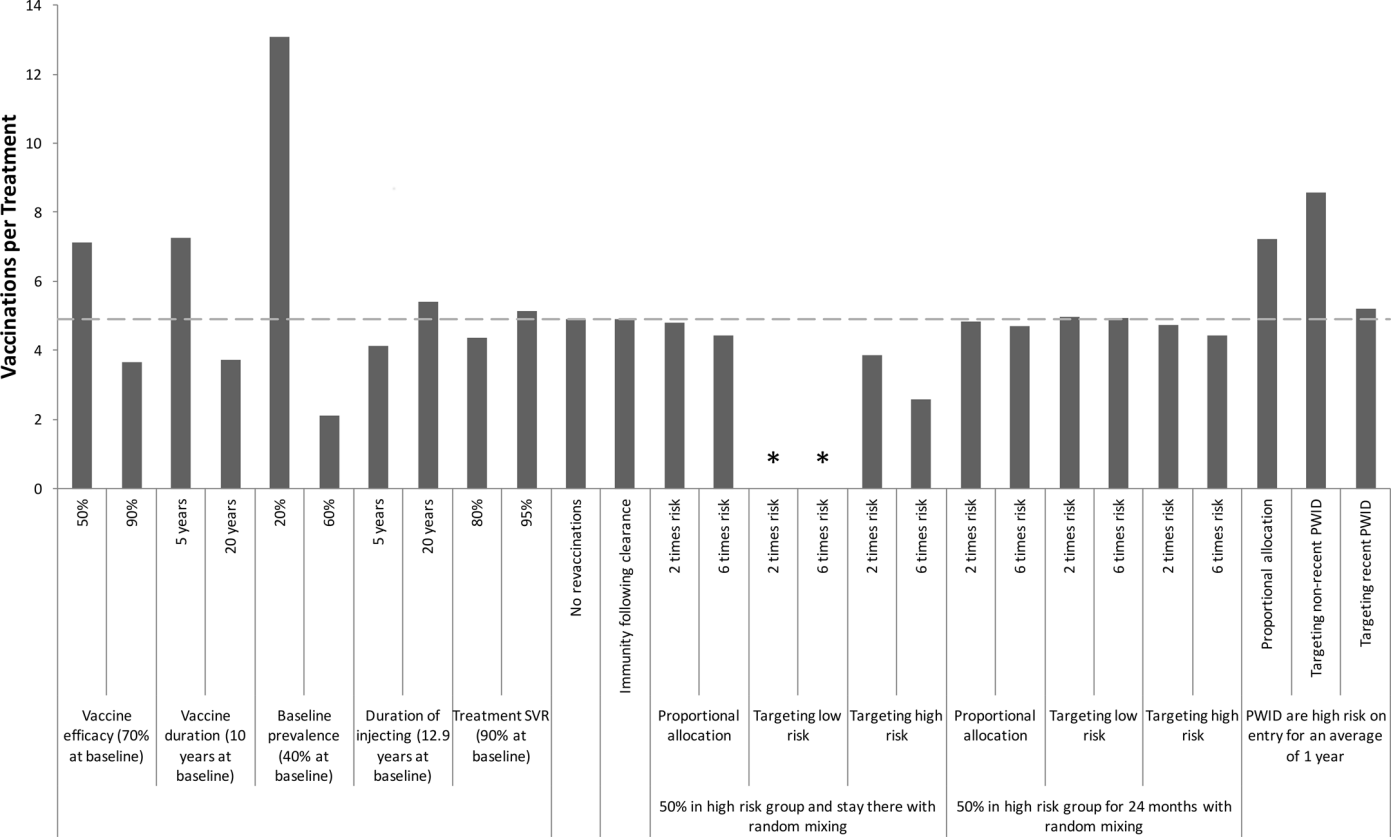
Sensitivity Analysis: Vaccination-to-treatment ratios for reducing HCV incidence. One-way sensitivity analyses on how specific changes in model parameters affect the ratio of the required annual vaccination to treatment rate needed to halve incidence **among PWID** over 40 years in the UK for a vaccine with 70% efficacy and duration of protection of 10 years. The baseline scenario (dashed line) assumed no behavioural risk heterogeneity with other baseline parameters shown in the figure. Asterisks mark scenarios in which the vaccine cannot halve incidence over 40 years.

In the baseline scenario for the moderate efficacy vaccine, the annual vaccination rates required to halve prevalence and incidence over 40 years were 7.6 and 4.9 times greater than the corresponding treatment rates, respectively. Figs 9 and 10 show that these ratios are sensitive to the vaccine’s efficacy or duration of protection, whilst changes in the treatment SVR have little effect (<11%). Baseline prevalence and injecting duration also have a considerable effect, with the vaccination-to-treatment ratios decreasing dramatically with HCV prevalence but increasing with injecting duration. Not allowing revaccination of PWID or assuming immunity following spontaneous clearance of infection have negligible impact (<1%) on the vaccination-to-treatment ratios.

Incorporating behavioural risk heterogeneity across all PWID has negligible effect unless vaccinations and treatments are risk targeted and PWID do not transition between risk groups. If this is the case, then targeting high-risk PWID reduces the vaccination-to-treatment ratios, whereas vaccinating low-risk PWID cannot halve prevalence or incidence over 40 years. Similarly, assuming recent PWID are higher-risk increases the vaccination-to-treatment ratios for halving prevalence and incidence irrespective of who is targeted.

## Discussion

Although regulators and manufacturers will likely strive for a high efficacy vaccine, our analysis suggests that even low efficacy HCV vaccines could have considerable impact; potentially halving HCV prevalence and incidence amongst PWID in 40 years for vaccine coverage levels comparable to what has been achieved for HBV amongst PWID in the UK, (72% in 2013[25]). Higher efficacy vaccines could achieve these impacts with lower vaccine coverage levels (25–56%) or over shorter time frames (20 years) and could achieve greater impact (75% reductions in HCV incidence and prevalence over 20 and 40 years respectively). Although these impact projections are favorable, our modeling suggests that similar reductions in HCV prevalence or incidence could be achieved with 4–16 or 2–11 times fewer treatments, respectively. Nonetheless, at current HCV drug costs and even reduced costs, these ratios suggest that vaccination could be a much cheaper strategy for reducing HCV transmission than scaling-up treatment[7, 8, 24].

To optimize impact and efficiency with HCV treatment and vaccination strategies, it is useful to understand when the benefits of vaccination are heightened. Importantly, our sensitivity analysis suggests that its relative benefits compared to treatment improve markedly in PWID populations with shorter injecting durations or higher HCV prevalences. Similarly, HCV vaccination compares better to treatment for reducing HCV incidence than reducing HCV prevalence, and when targeting higher-risk PWID[6]. This suggests that vaccination could be preferable to treatment in high prevalence settings or among high-risk PWID subgroups, when treatment alone may not be suitable because of the considerable risk of re-infection.

### Limitations

Several limitations need to be considered. Chief among these is the speculative nature of the vaccine characteristics. To counter this, we explored a range of efficacies and durations of protection to assess the effect of this uncertainty on our projections, and show that even low efficacy vaccines could have considerable prevention utility.

Our model was primarily parameterised to the UK, and therefore our findings may not be fully applicable to other settings. However, we performed extensive sensitivity analyses to show how our results may vary for settings with different HCV prevalence or injecting duration.

We did not consider the possibility of a boosted immune response amongst vaccinated PWID who are exposed to HCV (as occurs with other infections, e.g. pertussis[26]) to avoid further speculation and over-complicating the model. If exposure to HCV boosted the immune response, our projections would underestimate the impact of vaccination amongst PWID, especially for vaccines with short duration of protection and in higher incidence settings with longer duration of injecting. Our projections for the impact of vaccination should therefore be considered conservative, further enforcing the conclusion that vaccination would be favorable to treatment.

Lastly, we only explored the impact of vaccination or treatment on their own, and neglected the impact of other HCV prevention interventions such as OST and NSP. We do this to obtain simple projections of the likely impact of a future vaccine, and to compare these projections with what could be achieved with new HCV treatments. Indeed, if a HCV vaccine were available, it is highly likely that HCV prevention interventions and HCV treatment amongst PWID would continue. Nevertheless, modelling studies project that continuing current treatment rates with DAAs amongst PWID in the UK (4.1–27.4 per 1000 PWID) are unlikely to have measurable impact in the next 15 years without further treatment scale-up[27, 28].

### Other studies

Our results are consistent with previous modeling which suggested that HCV vaccination amongst PWID in San Francisco could substantially reduce the prevalence and incidence of chronic HCV amongst PWID, especially if targeted to high-risk PWID[29]. Our analysis adds additional insights on how the impact of vaccination may vary in different PWID settings, for different vaccination durations, and makes a comparison of vaccination with new treatments. Additionally, two cost-effectiveness studies have found that HCV vaccination of PWID in Brazil[30] and Canada[31] would be cost-effective compared to ribavirin and interferon. Although we did not undertake a cost-effectiveness analysis, our findings suggest that vaccination could be cheaper than treatment for achieving large reductions in HCV prevalence.

### Implications

Previous modeling has suggested that traditional prevention interventions (OST and NSP) are unlikely to reduce HCV prevalence to low levels without HCV treatment scale-up[6, 24]. Our analysis suggests that vaccination could also be an important intervention strategy.

The infrastructure for HCV vaccination is already in place in many settings through existing HBV vaccination programmes for PWID, including through NSPs[32], prisons[33] or drug treatment centres[34] as suggested by the World Health Organization[35]. Vaccinating in such settings can achieve high coverage amongst PWID. For instance, in the UK only people at high-risk of infection are vaccinated against HBV and high coverage amongst PWID (72% in 2013[25]) has been achieved due to vaccination programs in prison (where high incarceration rates amongst PWID[36, 37] provide an ideal opportunity to vaccinate PWID) and at drug services[38]. Additionally, small cash incentives can increase both the coverage and completion rate of HBV vaccination amongst PWID[39, 40]. This suggests that when available, HCV vaccination may be more easily scaled-up, especially in the UK, than HCV treatment which would require greater medical infrastructure and expertise due to the testing needed to assess treatment eligibility, progress and success[41]. Also, fewer follow-ups for a vaccination strategy may be necessary as some vaccines are partially effective even with a single dose. However, if multiple doses are required, this could reduce effectiveness; for example, the recombinant hepatitis B vaccine Engerix-B achieves seroprotection rates of only 5.4–20.4% after the first dose, with increased protection obtained after subsequent doses[42]. Hence, poor adherence to a vaccination schedule could limit the impact of a HCV vaccine. However, in 2014, 83% of PWID in Scotland who had received at least one dose of HBV vaccination had received all 3 doses[43], suggesting that a multi-dose schedule may not limit the impact of HCV vaccination in the UK. Furthermore, a booster dose may be required considerably later than the initial dose (e.g. 5 years). Although there is limited data to inform how PWID would adhere to such a schedule, it is likely that delays in receiving the booster dose could limit the impact of such a vaccine compared to a vaccine providing similar protection without needing a booster dose, as is modelled in our study.

Importantly, the costs of HCV vaccination are likely to be far lower than the current cost of HCV treatment in high-income countries. For instance, the cost of vaccinating PWID for HBV in a NSP or prison setting has been estimated to be less than $200 per PWID in the USA[44, 45]. Even if the HCV vaccine cost was towards the high end of currently available vaccines ($500 for the meningitis B vaccine, Bexsero) the cost per PWID would likely still be at least 50 times less than the current cost of HCV treatment in high income settings. Although the costs of HCV treatment are likely to decrease in coming years, the costs of HCV vaccination, when available, are still likely to be a fraction of the cost of treatment. Furthermore, although some lower and middle income countries are already purchasing the new HCV treatments at under $900 per 12 week course[9] as well as generic Sofosbuvir from as little as $483 per 12 week course[46], similar price differentials between developed and developing countries are also likely to apply to a vaccine. In addition, infrastructure issues for scaling-up HCV case-finding and treatment may be more challenging in these settings because of the poor level of healthcare available, further favoring the introduction of a HCV vaccine.

Lastly, there is little evidence of a protective immune response following treatment with DAAs, which may limit the use of treatment as a solitary prevention intervention, especially in high prevalence settings where reinfection is likely. However, since treatment and vaccination would be targeted to different PWID, it is possible that treatment and vaccination could act in unison, with HCV uninfected PWID receiving vaccination and HCV infected PWID receiving treatment, and possibly then vaccination.

## Supporting Information

S1 Fig Impact projections of HCV vaccination. Projected reduction in chronic prevalence among PWID in the UK (chronic prevalence = 40%) at 25 and 50 years as well as the maximum possible reduction that is achieved for various vaccination rates (Φ_v_ per 1000 PWID per year) for a low (50% protection for 5 years), moderate (70% protection for 10 years) and high efficacy vaccine (90% protection for 20 years).
(PDF)

S2 Fig Effect of duration of injecting on the impact of vaccination. The effect of duration of injecting on the annual vaccination rate (per 1000 PWID) required to halve HCV chronic prevalence among PWID in the UK over 40 years for vaccines with 70% protection over 2.5, 5, 10 and 20 years.
(PDF)

S3 Fig Effect of duration of protection on the impact of vaccination. The projected reduction in HCV chronic prevalence among PWID in the UK at 20 years achieved by vaccinating PWID at annual rates of 25, 50 and 100 per 1000 PWID with a 50, 70 and 90% vaccine as the duration of protection varies.
(PDF)

S1 File Model Equations. (DOCX)
