# Supplementary figures and images for: The Potential Impact of a Hepatitis C Vaccine for People Who Inject Drugs: Is a Vaccine Needed in the Age of Direct-Acting Antivirals?
Source: PLoS One. 2016 May 25;11(5):e0156213. doi: 10.1371/journal.pone.0156213 (PMC4880220; doi:10.1371/journal.pone.0156213)

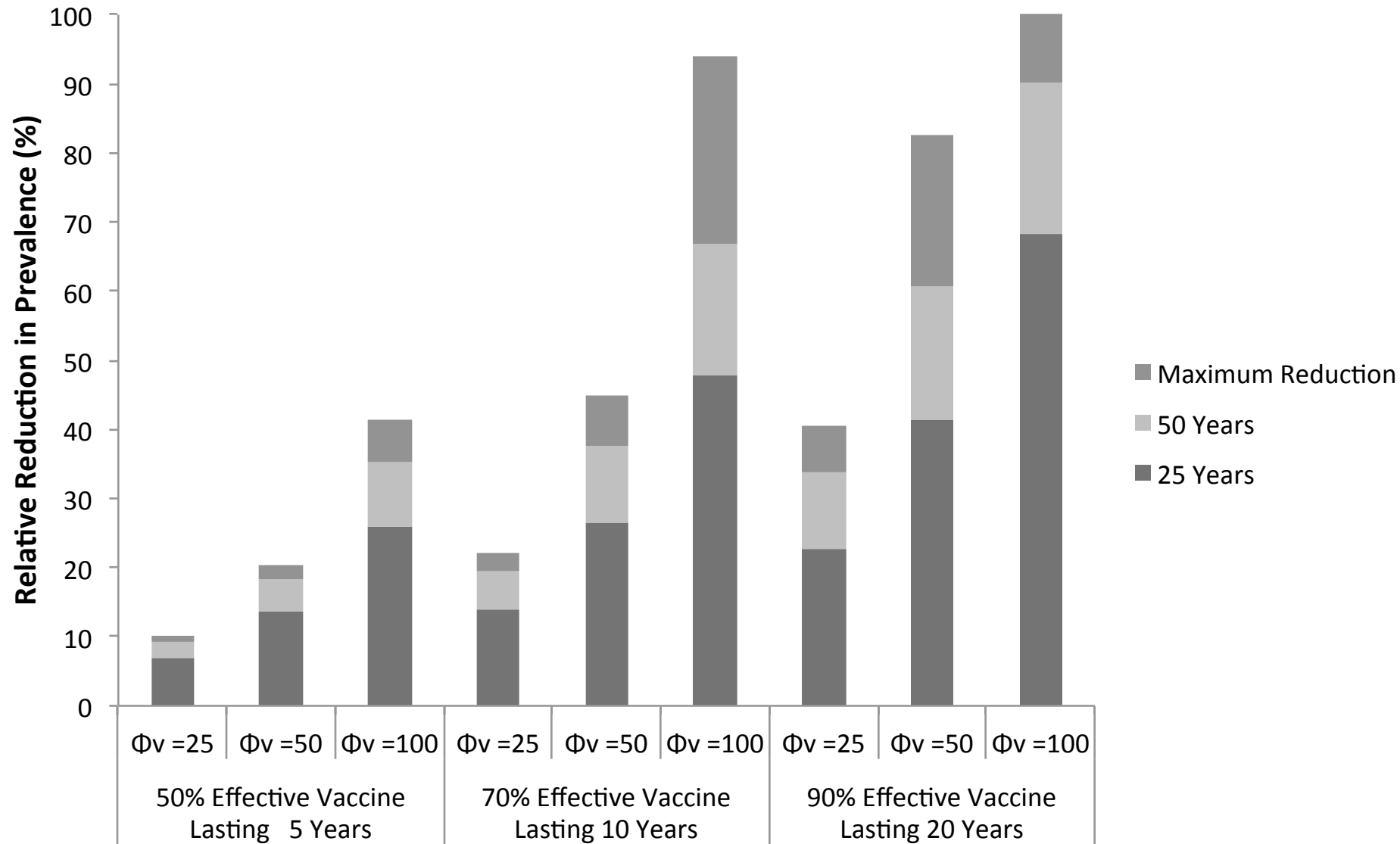

Supplement: S1 Fig — Projected reduction in chronic prevalence among PWID in the UK (chronic prevalence = 40%) at 25 and 50 years as well as the maximum possible reduction that is achieved for various vaccination rates (Φv per 1000 PWID per year) for a low (50% protection for 5 years), moderate (70% protection for 10 years) and high efficacy vaccine (90% protection for 20 years). (PDF) [file pone.0156213.s001.pdf]

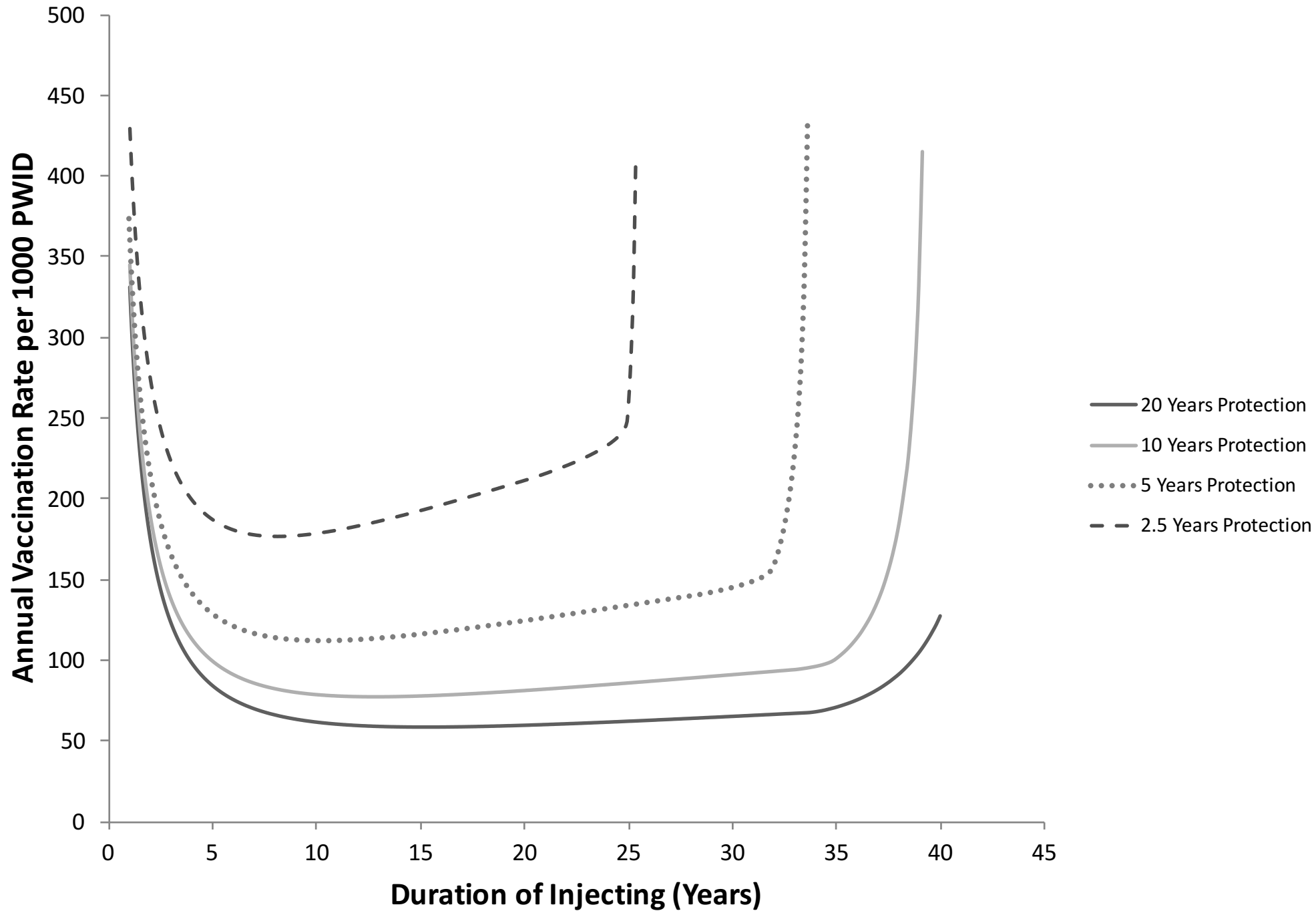

Supplement: S2 Fig — The effect of duration of injecting on the annual vaccination rate (per 1000 PWID) required to halve HCV chronic prevalence among PWID in the UK over 40 years for vaccines with 70% protection over 2.5, 5, 10 and 20 years. (PDF) [file pone.0156213.s002.pdf]

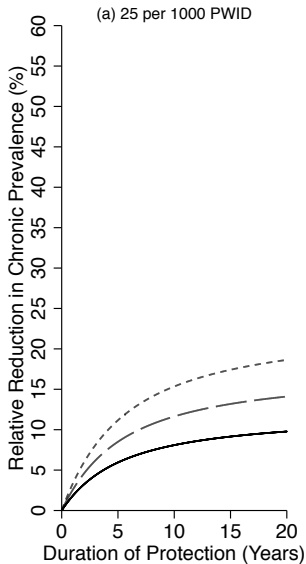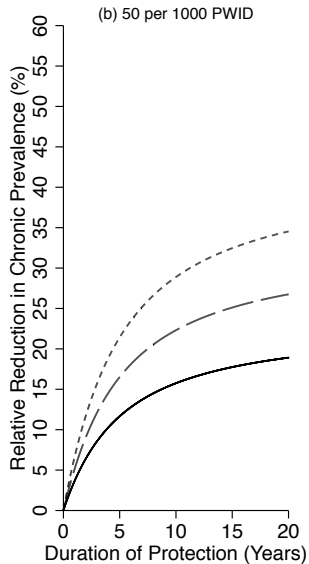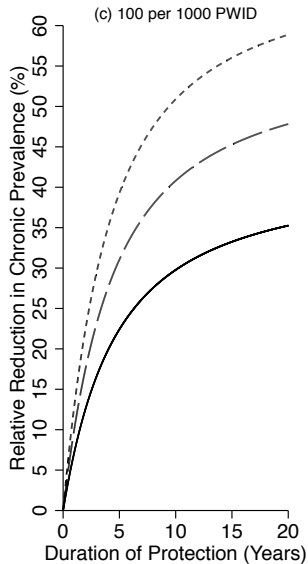

— 50% Protection      - - - 70% Protection  
- - - 90% Protection

Supplement: S3 Fig — The projected reduction in HCV chronic prevalence among PWID in the UK at 20 years achieved by vaccinating PWID at annual rates of 25, 50 and 100 per 1000 PWID with a 50, 70 and 90% vaccine as the duration of protection varies. (PDF) [file pone.0156213.s003.pdf]
