# Supplementary material for: The Potential Impact of a Hepatitis C Vaccine for People Who Inject Drugs: Is a Vaccine Needed in the Age of Direct-Acting Antivirals?
Source: PLoS One. 2016 May 25;11(5):e0156213. doi: 10.1371/journal.pone.0156213 (PMC4880220; doi:10.1371/journal.pone.0156213)
Supplement: S1 File — (DOCX) [file pone.0156213.s004.docx]

# Supplementary Materials

# Equations for the Baseline Vaccine Model

The model simulates HCV transmission amongst a population of active PWID comprised of three compartments, S denoting susceptible PWID, C denoting chronically infected PWID and V denoting PWID who currently have protection against transmission from a vaccine, t is the time in years and N is the total population size (N=S+C+V). The model equations are as follows:

$$\frac{\mathrm{dS}}{\mathrm{dt}} =\theta-\mu S- f\left( S \right)-\lambda\left( 1-\alpha\right)\frac{C}{N} S+\delta V$$

$$\frac{\mathrm{dC}}{\mathrm{dt}} = -\mu C+\lambda(1-\alpha)\frac{C}{N}(S+\eta V)$$

$$\frac{\mathrm{dV}}{\mathrm{dt}} = f(S) - (\mu+\delta) V -\eta\lambda(1-\alpha)\frac{C}{N} V$$

Where

$$f\left( S \right)= \min_{} (\Phi_{v}, S)$$

New PWID enter the susceptible pool at fixed rate θ and leave all compartments either through death or ceasing injection (rate μ). The model is dynamic, with the rate of infection of susceptible PWID being proportional to the current prevalence of chronic infection and the infection rate, λ. Acute infection spontaneously clears in a proportion, α, who then become susceptible again. The remaining infected fraction, (1 - α) progress to chronic infection. We neglect new infections from PWID who spontaneously clear the acute phase due to the short duration of this phase and the low proportion of PWID who clear this phase.

Susceptible (RNA-negative) PWID are vaccinated at a fixed rate (Φ_v_ per 1000 annually) unless the number of susceptible PWID is below Φ_v_, whereupon all susceptible PWID are vaccinated. PWID remain in the vaccinated state for an average duration 1\δ where they experience a factor η lower infection rate than susceptible PWID. This means 100(1-η)%, is the degree to which the vaccine reduces the risk of HCV acquisition or the vaccine efficacy. It is assumed that PWID can be revaccinated following loss of protection from the vaccine, providing equal levels of protection as PWID naïve to vaccination. The impact of having no revaccination is tested in the sensitivity analysis with the introduction of a 4^th^ compartment which denotes those susceptible PWID who have previously been vaccinated, which PWID enter after protection from vaccination is lost. They experience the same infection dynamics as susceptible PWID who have never been vaccinated.

# 2) Equations for the Baseline Treatment Model

The model for simulating the effect of HCV treatment of PWID is taken from [1], and simulates HCV transmission amongst a population of active PWID comprised of three compartments, S denoting susceptible PWID, C denoting chronically infected PWID and T denoting PWID who are currently receiving treatment, t is the time in years and N is the total population size (N=S+C+T).The model equations are as follows:

$$\frac{\mathrm{dS}}{\mathrm{dt}} =\theta-\mu S-\lambda(1-\alpha)\frac{C}{N}S +\omega\pi T$$

$$\frac{\mathrm{dC}}{\mathrm{dt}} = -\mu C -g(C) +\lambda(1-\alpha)\frac{C}{N}S +\omega(1-\pi) T$$

$$\frac{\mathrm{dT}}{\mathrm{dt}} = g(C) - (\mu+\omega)T$$

Where

$$g(C) =\min_{} (\Phi_{t},C)$$

In this model, the dynamics for entry, exit and infection are identical to the vaccine model and any differences with the vaccine model are due to the intervention compartments (T and V). PWID are enrolled onto treatment at a fixed rate (Φ_t_ per 1000 annually) unless the number of chronically infected PWID is below Φ_t_, whereupon all infected PWID are enrolled. PWID undergoing treatment are assumed not to be infective due to the rapid reduction in viral load when on treatment[2]. PWID leave treatment with rate $\omega$ with a proportion $\pi$being successfully treated. No immunity is assumed following successful treatment, with successfully treated PWID returning to the susceptible compartment where their rate of infection and rate of spontaneous clearance are equivalent to PWID naïve to treatment. As a consequence of the variety of treatments soon to be available, it is also assumed that PWID can be retreated after failed treatment without loss in sustained viral response (SVR).

# 3) Extended Models to Include Risk Heterogeneity

In the sensitivity analysis, the vaccine and treatment models were extended to include a stratification of PWID into low and high-risk groups. Two scenarios regarding transitions between these groups were considered. In the first, no transitions between the risk groups were allowed corresponding to PWID being either low or high-risk for the entirety of their injecting career. In the second, PWID remained high-risk for 24 months and then became low risk. These high-risk PWID were replaced by low-risk PWID from the susceptible, chronically infected and vaccinated compartments according to the proportions of PWID in each compartment. In the model equations, the rate of leaving the high-risk group was given by the parameter $\tau$, which was set to 0 for the first scenario and 0.5 for the second scenario where PWID are high-risk for only 24 months.

The proportion of PWID entering the low-risk group is given by $p_{low}$, which in the sensitivity analysis was set to 0.5, corresponding to the proportion of PWID injecting crack or currently homeless in the UK[3]. The transmission risk for high-risk PWID were assumed to be 2 or 6 ($\gamma$ in the model equations) times higher than for low-risk PWID corresponding to the elevated HCV acquisition risk amongst PWID who inject crack or are homeless [3, 4]. For each risk factor,$\gamma,$ considered, the infection rate, λ, was adapted so that the total chronic prevalence across all PWID was still 40% as in the baseline scenario. PWID mixed randomly due to the minimal effect moderate like-to-like mixing has had in previous analyses. [4-6]

### Extended Vaccine Model to Include Risk Heterogeneity

The equations for the vaccine model are given below, where $S_{low}$, $C_{low}$ and $V_{low}$ are the number of PWID in the low-risk group which are susceptible, chronically infected and vaccinated respectively; $S_{high}, C_{high}$ and $V_{high}$are similarly the number of susceptible, chronically infected and vaccinated high-risk PWID; and $N_{low}$and $N_{high}$are the total number of PWID that are low-risk and high-risk respectively:

For low-risk IDU:

$$\frac{dS_{low}}{dt}= p_{low}\theta-\mu S_{low}-f\left( S_{low} \right)+\tau S_{high}-\tau N_{high}\frac{S_{low}}{N_{low}}-\lambda\left( 1-\alpha\right)\left[ \frac{C_{low}+\gamma C_{high}}{N_{low}+\gamma N_{high}} \right] S_{low} +\delta V_{low}$$

$$\frac{dC_{low}}{dt} = -\mu C_{low}+\tau C_{high}-\tau N_{high}\frac{C_{low}}{N_{low}}+\lambda\left( 1-\alpha\right)\left[ \frac{C_{low}+\gamma C_{high}}{N_{low}+\gamma N_{high}} \right](S_{low} +\eta V_{low})$$

$$\frac{dV_{low}}{dt} = f(S_{low}) - \left( \mu+\delta\right)V_{low}+\tau V_{high}-\tau N_{high}\frac{V_{low}}{N_{low}} -\eta\lambda\left( 1-\alpha\right)\left[ \frac{C_{low}+\gamma C_{high}}{N_{low}+\gamma N_{high}} \right]V_{low}$$

For high-risk PWID:

$$\frac{dS_{high}}{dt} = \left( 1 - p_{low} \right)\theta-\left( \mu+\tau\right)S_{high} - f(S_{high})+\delta V_{high} +\tau N_{high}\frac{S_{low}}{N_{low}}-\gamma\lambda\left( 1-\alpha\right)\left[ \frac{C_{low}+\gamma C_{high}}{N_{low}+\gamma N_{high}} \right]S_{high}$$

$$\frac{dC_{high}}{dt}=\gamma\lambda\left( 1-\alpha\right) \left[ \frac{C_{low}+\gamma C_{high}}{N_{low}+\gamma N_{high}} \right]\left( S_{high} +\eta V_{high} \right)+\tau N_{high}\frac{C_{low}}{N_{low}}-\left( \mu+\tau\right)C_{high}$$

$$\frac{dV_{high}}{dt} = f\left( S_{high} \right)- \left( \mu+\tau+\delta\right)V_{high} +{\tau N}_{high}\frac{V_{low}}{N_{low}} -\eta\gamma\lambda\left( 1-\alpha\right)\left[ \frac{C_{low}+\gamma C_{high}}{N_{low}+\gamma N_{high}} \right]V_{high}$$

Three different vaccination allocations were considered:

1) vaccination of low-risk PWID only, which can be considered the worst case scenario as those least at risk of infection are gaining vaccine protection

2) vaccination of high-risk PWID only, the best case scenario as those most at risk of infection are gaining vaccine protection

3) untargeted vaccination where both low and high-risk PWID are vaccinated proportionately according to the number of susceptible PWID within each risk group.

These vaccination strategies are captured in the model equations by $f\left( S_{low} \right)$ and $f\left( S_{high} \right)$ which are defined as follows:

$$f\left( S_{low} \right)=\left\{ \begin{matrix} \min_{} (\Phi, S_{low}) & \text{for vaccination of low-risk PWID} \\ 0 & \text{for vaccination of high-risk PWID} \\ \min_{} \left( \frac{S_{low}}{S_{low}+S_{high}}\Phi, S_{low} \right) & \text{for random vaccination} \end{matrix} \right.$$

and

$$f\left( S_{high} \right)=\left\{ \begin{matrix} 0 & \text{for vaccination of low-risk PWID} \\ \min_{} (\Phi, S_{high}) & \text{for vaccination of high-risk PWID} \\ \min_{} \left( \frac{S_{high}}{S_{low}+S_{high}}\Phi, S_{high} \right) & \text{for random vaccination} \end{matrix} \right.$$

### Extended Treatment Model to Include Risk Heterogeneity

The equations for the extended treatment model are given below, where $S_{low}$, $C_{low}$ and $T_{low}$ are the number of PWID in the low-risk group which are susceptible, chronically infected and in treatment respectively; $S_{high}$, $C_{high}$and $T_{high}$ are similarly the number of susceptible, chronically infected and in treatment high-risk PWID; and $N_{low}$ and $N_{high}$ are the total number of PWID that are low-risk and high-risk respectively:

For low-risk PWID:

$$\frac{dS_{low}}{dt}= p_{low}\theta-\mu S_{low} -\lambda\left( 1-\alpha\right)\left[ \frac{C_{low}+\gamma C_{high}}{N_{low}+\gamma N_{high}} \right]S_{low} +\tau S_{high} -\tau N_{high}\frac{S_{low}}{N_{low}}+\omega\pi T_{low}$$

$$\frac{dC_{low}}{dt}= -\mu C_{low} - g\left( C_{low} \right)+\tau C_{high} -\tau N_{high}\frac{C_{low}}{N_{low}}+\omega\left( 1-\pi\right)T_{low}+\lambda\left( 1-\alpha\right)\left[ \frac{C_{low}+\gamma C_{high}}{N_{low}+\gamma N_{high}} \right]S_{low}$$

$$\frac{dT_{low}}{dt}= g\left( C_{low} \right)- \left( \mu+\omega\right)T_{low} +\tau T_{high} -\tau N_{high}\frac{T_{low}}{N_{low}}$$

For high-risk PWID:

$$\frac{dS_{high}}{dt} = \left( 1 - p_{low} \right)\theta-\left( \mu+\tau\right)S_{high} +\delta V_{high} +\tau N_{high}\frac{S_{low}}{N_{low}}-\omega\pi T_{high}+\gamma\lambda\left( 1-\alpha\right)\left[ \frac{C_{low}+\gamma C_{high}}{N_{low}+\gamma{}_{high}} \right]S_{high}$$

$$\frac{dC_{high}}{dt} = -g\left( C_{high} \right)+\gamma\lambda\left( 1-\alpha\right) \left[ \frac{C_{low}+\gamma C_{high}}{N_{low}+\gamma N_{high}} \right]S_{high} +\tau N_{high}\frac{C_{low}}{N_{low}}-\left( \mu+\tau\right) C_{high}+\omega\left( 1-\pi\right)T_{high}$$

$$\frac{dT_{high}}{dt} = g\left( C_{high} \right)- \left( \mu+\tau+\omega\right)T_{high}+\tau N_{high}\frac{V_{low}}{N_{low}}$$

Similar to the vaccine model, three different treatment allocations were considered:

1) treatment of low-risk PWID only, which can be considered the worst case scenario as those who are least likely to infect other PWID are treated.

2) treatment of high-risk PWID only, the best case scenario as those who are most likely to infect other PWID are treated.

3) random treatment where both low and high-risk PWID are treated proportionately according to the number of chronically infected PWID within each risk group.

These treatment strategies are represented in the model equations by $g(C_{low})$ and $g(C_{high})$ which are defined as follows:

$$g\left( C_{low} \right)=\left\{ \begin{matrix} \min_{} (\Phi, C_{low}) & \text{for treatment of low-risk PWID} \\ 0 & \text{for treatment of high-risk PWID} \\ \min_{} \left( \frac{C_{low}}{C_{low}+C_{high}}\Phi, C_{low} \right) & \text{for random treatment} \end{matrix} \right.$$

and

$$g\left( C_{high} \right)=\left\{ \begin{matrix} 0 & \text{for treatment of low-risk PWID} \\ \min_{} (\Phi, C_{high}) & \text{for treatment of high-risk PWID} \\ \min_{} \left( \frac{C_{high}}{C_{low}+C_{high}}\Phi, C_{high} \right) & \text{for random treatment} \end{matrix} \right.$$

**High-risk for first year**

A final sensitivity analysis considered the scenario where PWID are high-risk on initiation of injecting and remain high-risk for an average of one year. After a year of injecting PWID become lower risk where they remain for the remainder of their injecting careers. This scenario requires a slight modification of the above model equations so that low-risk PWID do not transition to high-risk to match the flow of high-risk recent PWID into low-risk. Furthermore, since all PWID enter the model as high-risk, p_high_ must be set to 1. The infection rate, λ, and risk factor,$\gamma$, were chosen so that the total endemic prevalence without interventions was 40% with the chronic prevalence among recent PWID (within their first year) set to be half that of non-recent PWID[7].

# References

1. Martin NK, Vickerman P, Hickman M. Mathematical modelling of hepatitis C treatment for injecting drug users. Journal of theoretical biology. 2011;274(1):58-66. doi: 10.1016/j.jtbi.2010.12.041. PubMed PMID: 21236265.

2. Gane EJ, Stedman CA, Hyland RH, Ding X, Svarovskaia E, Symonds WT, et al. Nucleotide polymerase inhibitor sofosbuvir plus ribavirin for hepatitis C. The New England journal of medicine. 2013;368(1):34-44. doi: 10.1056/NEJMoa1208953. PubMed PMID: 23281974.

3. Turner KM, Hutchinson S, Vickerman P, Hope V, Craine N, Palmateer N, et al. The impact of needle and syringe provision and opiate substitution therapy on the incidence of hepatitis C virus in injecting drug users: pooling of UK evidence. Addiction. 2011;106(11):1978-88. doi: 10.1111/j.1360-0443.2011.03515.x. PubMed PMID: 21615585.

4. Vickerman P, Martin N, Turner K, Hickman M. Can needle and syringe programmes and opiate substitution therapy achieve substantial reductions in hepatitis C virus prevalence? Model projections for different epidemic settings. Addiction. 2012;107(11):1984-95. doi: 10.1111/j.1360-0443.2012.03932.x. PubMed PMID: 22564041.

5. Martin NK, Hickman M, Hutchinson SJ, Goldberg DJ, Vickerman P. Combination interventions to prevent HCV transmission among people who inject drugs: modeling the impact of antiviral treatment, needle and syringe programs, and opiate substitution therapy. Clinical infectious diseases : an official publication of the Infectious Diseases Society of America. 2013;57 Suppl 2:S39-45. doi: 10.1093/cid/cit296. PubMed PMID: 23884064; PubMed Central PMCID: PMC3722076.

6. Martin NK, Vickerman P, Grebely J, Hellard M, Hutchinson SJ, Lima VD, et al. Hepatitis C virus treatment for prevention among people who inject drugs: Modeling treatment scale-up in the age of direct-acting antivirals. Hepatology. 2013;58(5):1598-609. doi: 10.1002/hep.26431. PubMed PMID: 23553643; PubMed Central PMCID: PMC3933734.

7. Hagan H, Pouget ER, Des Jarlais DC, Lelutiu-Weinberger C. Meta-regression of hepatitis C virus infection in relation to time since onset of illicit drug injection: the influence of time and place. American journal of epidemiology. 2008;168(10):1099-109. doi: 10.1093/aje/kwn237. PubMed PMID: 18849303; PubMed Central PMCID: PMC2727245.
